# Do I care for you or for me? Processing of protected and non-protected moral values in subjects with extreme scores on the Dark Triad

**DOI:** 10.1007/s00406-022-01489-3

**Published:** 2022-10-08

**Authors:** Kai Ueltzhöffer, Corinna Roth, Corinne Neukel, Katja Bertsch, Friederike Nüssel, Sabine C. Herpertz

**Affiliations:** 1 Department of General Psychiatry, Center for Psychosocial Medicine, Heidelberg University, Voßstraße 2, 69115 Heidelberg, Germany; 2 German Cancer Center, Heidelberg University, 69117 Heidelberg, Germany; 3 The Wellcome Centre for Human Neuroimaging, University College London, London, WC1N 3AR UK; 4 Institute of Clinical Psychology and Psychotherapy, LMU München, 80802 Munich, Germany

**Keywords:** Dark triad, Moral values, Functional MRI, Script-based imagery, Protected values

## Abstract

Protected moral values facilitate empathic concern for others, who are exposed to an existential threat, so that one spontaneously helps without taking into account utilitarian cost–benefit considerations. Subjects scoring high on the “Dark Triad” machiavellism, psychopathy, and narcissism are prone to ignore such appeals for selfless help. Until now, data on moral processing and moral decision-making following requests for altruistic help, which directly contrast appeals to protected and non-protected values in subjects with high and low scores on Dark Triad traits, have been missing. In this pilot study 25 healthy subjects with high and 27 with low Dark Triad scores participated in this functional magnetic resonance imaging study. We used a script-driven imagery paradigm to directly contrast requests for selfless help appealing to protected versus non-protected, negotiable moral values. Appeals to protected versus non-protected moral values elicited stronger activations in a large network including insula, amygdala, supramarginal gyrus, and dorsolateral prefrontal cortex. Non-protected values evoked stronger activation in superior frontal sulcus, occipito-temporal junction, and posterior cingulate cortex. During decision-making, high-scorers on the Dark Triad showed increased activations in the superior parietal lobule, precuneus, and intraparietal sulcus. Behaviorally, protected versus non-protected values strongly reduced the reliance on personal cost–benefit calculations in low-scorers, while high-scorers continued to rely on utilitarian deliberations. Data suggest that appeals to protected versus non-protected values activate distinct brain regions associated with strong moral emotions, other-directed cognition, and rule-based decision-making processes. High-scorers display an increased reliance on cost–benefit calculations, which persists even when protected values are threatened.

**Supplementary Information:**

The online version contains supplementary material available at 10.1007/s00406-022-01489-3.

## Introduction

It is not uncommon for people to risk their own material or physical wellbeing in order to help others, solely based on a sense of morality. Neuroscientific and empirical research has grounded this theoretical and philosophical construct in terms of biological function and neuronal implementation [1–4].

When studying moral values, one can distinguish protected from non-protected moral values [5, 6]. By definition, protected values supervene utilitarian cost–benefit calculations, invoke fixed directives for behavior, resist trade-offs with other values, and are to be followed at any cost. People are concerned about consequently acting according to protected values, rather than just about the final consequences of their behavior [5]. Protected values are deeply embedded into the cultural practices, narratives, and identity of groups and individuals sharing these values, which is why they are also known as “sacred” values [6–8]. Accordingly, protected values do not allow any compromise [7]. Questioning protected values means attacking what is 'valuable' and functional for personal and social identity [9]. They are tied to strong moral emotions [10] and have a deep rooted biological basis including phylogenetically old brain circuits involved in salience processing in the anterior insula and amygdala, as well as brain circuits implementing social cognition, such as the posterior superior temporal gyrus, angular gyrus, and temporo-parietal junction [11, 12]. Furthermore, neuroimaging studies of decision-making based on protected values report activation of executive cognitive control networks, including dorsolateral and ventrolateral prefrontal cortices [13, 14], in accordance with the rule-based nature of moral decisions. In contrast, non-protected values are negotiable: Although behavior in accordance with non-protected moral values is positively sanctioned in social groups, these are less fixed in their implication for action and invoke utilitarian cost–benefit calculations [13, 14]. In a neuroimaging study comparing protected to non-protected values, the latter were found to more intensely activate the posterior medial cortex and the left temporo-parietal junction [14]. Even within the same social and cultural context, the way in which individuals respond to appeals to moral values can differ substantially, and these interindividual differences are stable across different environmental contexts [15]. A subgroup especially prone to ignore appeals to moral values are people scoring high on Dark Triad trait measures [16]. The Dark Triad includes three conceptually different, but empirically overlapping personality variables, namely machiavellianism, psychopathy, and narcissism [17]. Jonason et al. [18] demonstrated correlations between any subscale of the Dark Triad and the tendency to strongly cherish one’s own interests. They concluded that Dark Triad traits share “a unique complex of values that might run counter to societal expectations for selflessness” [18]. Therefore, persons scoring high on the Dark Triad often present challenges to their social groups as they tend to put their own needs before the needs of others. Current societal crises, such as the COVID-19 pandemic, the Ukraine war (and related discussions about the economic embargo against Russia) or the climate disaster challenge the almost universal protected value of cherishing and protecting the health and life of others even at the cost of one’s own benefits. Individual differences in personality related to the Dark Triad, namely degree of selfishness, callousness and empathic concern toward others, apparently influence decision-making that impacts this protected value [19].

Until now, there has been no study on differential processing of appeals for selfless help in contexts, in which protected versus non-protected values are threatened, in subjects with high and low scores on the Dark Triad. Thus, the present functional resonance imaging study examines behavior and neural correlates of considering moral values and moral decision-making based on imagined ecologically valid appeals to selflessly help a second person.

In line with prior work, we expected protected moral values to increase the willingness to help, decision confidence, and emotional involvement, and decrease the readiness to change the decision for any amount of money, compared to non-protected values. Furthermore, we expected narratives appealing to protected values to elicit stronger activations in regions of the salience network, such as the amygdala and the insula, in line with stronger elicited moral emotions. Decision-making based on protected values compared to non-protected values should correlate with increased activations in regions associated with the executive, rule-based control of behavior, moral and social cognition and decreased activations in regions associated with utilitarian cost–benefit calculations and subjective value.

In line with a common core of the Dark Triad in terms of callousness and cherishing oneself over others, we expected a decreased willingness to help, decreased emotional involvement, and an increased readiness to change the decision for some amount of money in high-scorers, compared to low-scorers. Furthermore, we expected decreased activations in areas associated with social and moral cognition, salience, moral emotions, and rule-based decision making, and increased activations in areas involved in utilitarian cost–benefit calculations, self-related cognition and subjective value. Crucially, we hypothesized that dark triad scores would modulate the differential processing of protected versus non-protected moral values. Given the common core of the dark triad traits in terms of callousness and putting oneself before others, we expected a less pronounced effect of value type in high-scoring participants, both in terms of behavior and neural activity. Concretely, we expected a sustained reliance on utilitarian cost–benefit evaluations and only marginal increases in elicited moral emotions and salience during appeals to protected versus non-protected moral values in subjects scoring high on the dark triad versus subjects scoring low on the dark triad.

## Methods

### Participants

Male, right-handed participants, in the age range from 18 to 60 years, with no history of psychiatric or neurological disease, were recruited by local and online advertisements in the Heidelberg area. 264 participants initially completed a set of online questionnaires, including the Short Dark Triad with its subscales machiavellianism, narcissism, and psychopathy (SD3, [20]), and the Moral Competence Test (MCT, [21]). Chronbach’s alpha in our study were as follows: SD3 (sum): 0.947; SD3 Psychopathy: 0.886, SD3 Narcissism: 0.857, SD3 Machiavellianism: 0.876. Participants, whose SD-3 score were within the lowest or highest quartile of the sample, were contacted to perform a telephone screening where above exclusion criteria and MRI contraindications were checked. During the interview, avoidant, narcissistic, anti-social and borderline subscales of the International Personality Disorder Examination (IPDE, [22]) were administered, to screen for undiagnosed personality disorders.

The final sample consisted of 27 participants scoring within the lowest (low-scorers, SD3 sum score < 56) and 25 participants scoring within the highest (high-scorers, SD3 sum score > 84) quartile of the SD3 in this sample, with the initial recruiting goal being 25 participants in each group. Descriptive statistics of the sample are shown in Table 1. The MCT competence (*C*-) score was used to ensure that the high- and low-scoring groups did not differ significantly in their cognitive ability to judge the moral quality of an argument. As the groups differed significantly with respect to their age, we included age as a subject-level covariate of no interest in all further analyses. All participants provided written informed consent for the protocol approved by the Ethics Committee of the Medical Faculty of Heidelberg University, Heidelberg, Germany, and were reimbursed for their participation.

**Table 1:** Sample description

	Low-scorer (*n* = 27)^a^		High-scorer (*n* = 25)^a^		Group difference	
	Mean	Std	Mean	Std	*T*	*p*
SD3 sum	51.8	3.3	90.3	6.5	− 27.1	< 0.001*
SD3 psychopathy	11.5	1.3	24.5	4.4	− 14.5	< 0.001*
SD3 narcissm	21.8	3.5	33.0	4.6	− 10.0	< 0.001*
SD3 machiavellianism	18.5	3.4	32.8	4.0	− 13.9	< 0.001*
*C*-index^b^	32.47	15.24	23.51	19.99	1.83	0.074
Education^c^	13.48	1.25	13.32	0.74	0.56	0.58
Age^d^	26.74	6.30	22.88	2.68	2.84	0.007*

*SD3* Short Dark Triad

^a^Participants in the lowest vs. highest quartile of an initially recruited sample, with respect to their score in the “short Dark Triad” questionnaire

^b^Competence score in the moral competence test

^c^Years of education

^d^In years

*p* < 0.05

### Script-driven imagery paradigm

**Fig. 1:**
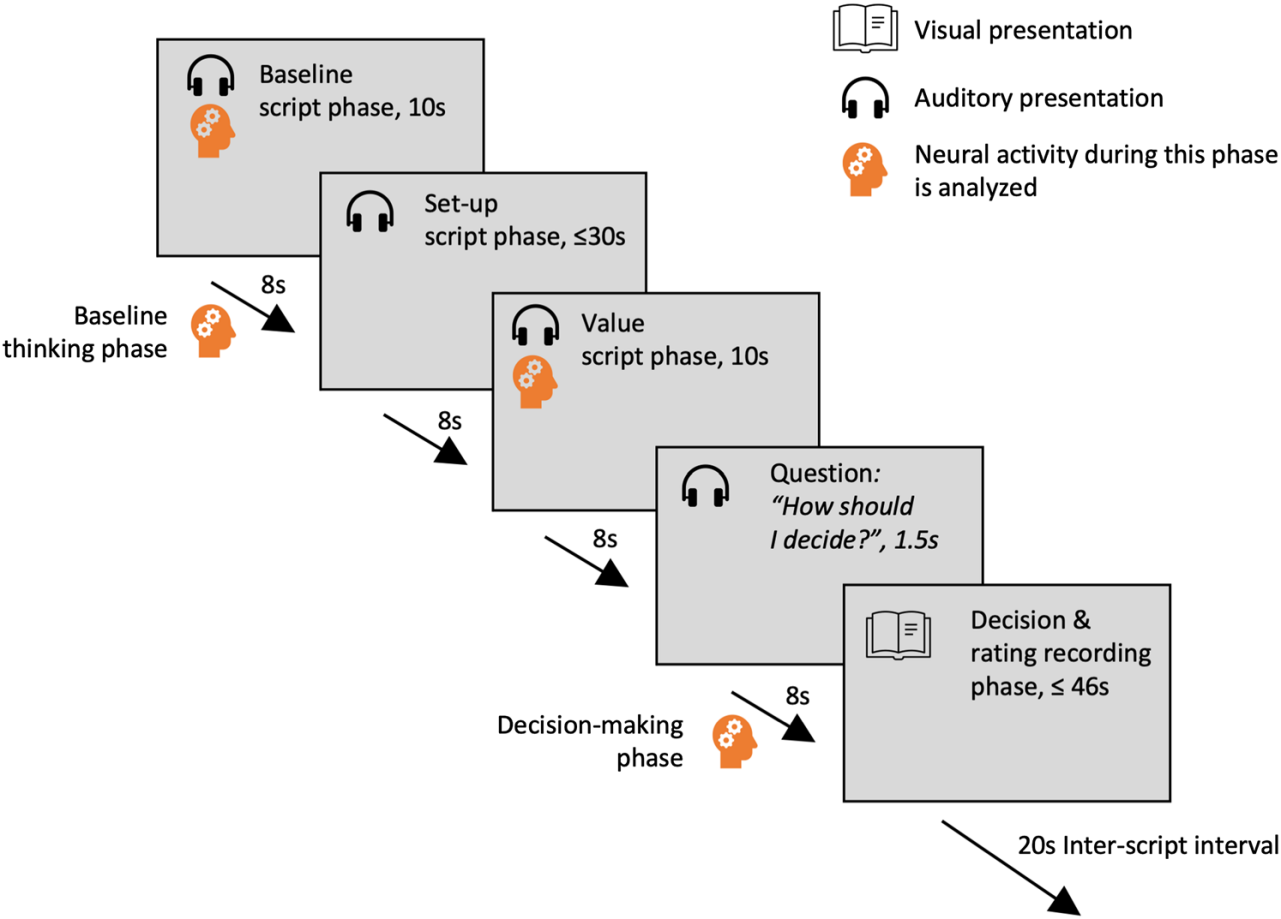
Schematic depiction of a single narrative. Each narrative consists of the following phases: (1) A phase outlining a neutral baseline situation (“baseline script phase”). (2) A set-up phase, outlining the context and the personal stakes of the participant in the following moral dilemma (“set-up script phase”). (3) A moral dilemma, in which the participant is appealed to help a second person, thereby incurring some cost by himself (e.g., a monetary cost, missing an important flight or appointment; “value script phase”). (4) The question, “How should I decide?”. (5) A decision and rating recording phase. Script phases are separated by an 8 s inter-phase interval, and successive scripts are separated by a 20 s inter-script interval

All narratives told a complete, ecologically valid, plausible story. They were narrated by a professional male actor in a neutral tone and steady pace, and in a first-person perspective, to foster perspective taking, absorption, vivid imagery and identification with the narratives [23]. Eight modular scripts were developed, as shown in Fig. 1. Each script contained a baseline script phase, outlining a neutral situation, a set-up script phase, outlining the personal stakes of the participant, a value script phase, presenting a request for selfless help by a second person, the question “How should I decide?”, and a decision and rating recording phase, where participants answered the following questions: (a) How they would act in the given situation. (b) How confident they were in their decision (visual analog scale from 0, “not at all”, to 100, “very much”). (c) If they would change their decision for any amount of money. (d) How emotionally involved they were with the presented situation (visual analog scale from 0, “not at all”, to 100, “very much”). The contrasted script phase, namely the baseline script phase and value script phase, were always 10 s long. The set-up phases were approximately 30 s long, the question “How should I decide?” was 1.5 s long. Inter-phase intervals were always 8 s long. Thus, including the inter-phase intervals, the narratives were always about 75.5 s long.

To directly contrast protected and non-protected values, we developed modular narratives, which were identical with respect to the baseline script phase, and the set-up script phase, but differed in the dilemma outlined in the value script phase, in terms of the nature of the appealed moral value: A second person was either depicted in face of an existentially threatening situation (“protected value script phase”), which could be averted by helping them. In this case, the narrative would appeal to a protected value. Alternatively, the second person was depicted in a position, where they required help to reach a certain goal, but were not immediately threatened (“non-protected value script phase”). In this case, the narrative would appeal to a non-protected value. This allowed to directly contrast the effects of the type of moral value (protected vs. non-protected), while keeping the rest of the imagined scene, context, and the associated verbal presentation, exactly the same. Each narrative was presented in these two versions, making up for a total of 16 narratives (example see Box 1).

Preliminary versions of the narratives were presented to 30 participants, recruited from Heidelberg University’s student body. These participants were asked to rate: (1) If most other people would help. (2) If these people would change their decision for any amount of money. (3) How guilty and (4) ashamed they would feel, if they had decided not to help. Based on these ratings, narratives were selected, where > 90% of the participants would not change their decision in the protected case, and > 50% of the participants would change their decision in the non-protected case. For all of the presented narratives, the protected versions were consistently rated to elicit higher feelings of shame and guilt, if one would decide to deny help, compared to their non-protected counterpart.

The experiment consisted of 2 blocks of 8 presented narratives each, separated by a short break. Participants were instructed to imagine the presented narratives as vividly as possible, as if they would just experience the narrated situations. The 16 narratives were presented in pseudo-randomized order, enforcing the constraints that no more of 3 consecutive narratives should appeal to the same type of moral value, and that narratives sharing the same baseline and set-up phase should not directly follow each other.

Individual phases of a narrative were separated by an 8 s inter-phase interval, and successive scripts were separated by a 20 s inter-script interval. We used the 8 s inter-phase interval following the baseline script phase as “baseline thinking phase”, and we analyzed the 8 s inter-phase interval following the question of how to decide as “decision-making phase”.

### Box 1: Example of a protected and non-protected script

Script appealing to a protected value:

*Baseline script phase:* I’m arriving at the airport. After working for months without a break, I’m looking forward to take off on a well-earned, two-week holiday

*Set-up script phase:* I’ve been saving for this trip for a long time. There will be no other opportunity – rebooking is impossible. Because I’m late, I fear I might be missing my flight. This would mean that my dream holiday would be cancelled. As I finally find a spot in the parking garage, only a few minutes are left until the boarding gate will close. I’m running as fast as I can across the deserted parking deck

*Protected value script phase:* Close to the exit, I spot a young child, lying on the floor. The child is bleeding heavily from a head wound, and is not reacting, as I start yelling at her

*Question:* How should I decide?

Script appealing to a non-protected value:

*Baseline script phase:* I’m arriving at the airport. After working for months without a break, I’m looking forward to take off on a well-earned, two-week holiday

*Set-up script phase:* I’ve been saving for this trip for a long time. There will be no other opportunity – rebooking is impossible. Because I’m late, I fear I might be missing my flight. This would mean that my dream holiday would be cancelled. As I finally find a spot in the parking garage, only a few minutes are left until the boarding gate will close. I’m running as fast as I can across the deserted parking deck

*Non-protected value script phase:* Close to the exit, a woman is approaching me. She asks, if I could show her the way to her gate – it would be important

*Question:* How should I decide?

### Statistical analysis of behavioral data

To explore the influence of group and value on the dependent variables “decision” (decision to follow or ignore the request for help), “change” (willingness to change the decision for any amount of money), confidence in the decision, and emotional involvement in the narrative, multi-level linear models were set up for each response variable. Logistic regression was used for the categorical outcome variables decision and change, and least squared errors regression was used for the scale variables confidence and emotional involvement. Group, value type, and their interaction were used as predictive variables, and all models included age as additional predictor, to control for potential confounding influences between age and group variables. A second-level variable represented the subject identity, to accommodate the nested structure of our data. We used a non-parametric bootstrapping procedure to derive confidence bounds for the parameter estimates (10,000 random samples), and random permutations of the group and value labels to derive a non-parametric null distribution (10,000 random samples). The Benjamini–Hochberg procedure with a conservative false-discovery rate of 0.05 was applied to control for multiple comparisons.

For three narratives (distributed over two low-scorers and one high-scorer), no response for the question: “Would you change your decision for any amount of money?” was recorded. These trials were excluded from the analysis of this behavioral variable.

Multi-level models were implemented, and behavioral results were visualized in the R statistical programming language, using functionality provided by the “haven”, “lme4”, “emmeans”, “ggplot2”, “ggpubr”, and “dplyr” packages (R Core Team, 2021).

### Analysis of MRI data

#### Acquisition and preprocessing of MRI data

During the experiment, imaging data were acquired in a 3 T Tim Trio whole-body scanner (Siemens, Erlangen, Germany), using a 32-channel head coil. Each T2* weighted functional volume consisted of forty axial slices. The parameters of the gradient echo planar imaging sequence used were: TR 2.34 s, TE 26 ms, voxel-size 2.3 × 2.3 × 2.3 mm^3^. Additionally, a T1 weighted, sagitally sliced anatomical images was recorded, featuring an isotropic resolution of 1 mm [3], using a magnetization prepared rapid gradient echo (MPRAGE) sequence.

To account for T1 saturation effects, the first 5 functional volumes of each participant were discarded. Functional MRI data were preprocessed using SPM12 (https://www.fil.ion.ucl.ac.uk/spm/software/spm12/) and MATLAB R2012b (The MathWorks, Natick, MA, USA). The individual functional images were realigned and resliced using the SPM12 default settings. The T1-weighted anatomical image was co-registered with the resulting mean functional image. The registered anatomical image was segmented into grey and white matter components using a unified segmentation approach [24] based on a tissue probability map in MNI space with 1.5 × 1.5 × 1.5 mm^3^ resolution, yielding normalization transformations from individual subject anatomy into MNI space. These transformations were then applied to the coregistered functional images and the normalized images were resliced to 2 × 2 × 2 mm^3^ resolution. The normalized functional images were smoothed using a Gaussian kernel of 8.0 mm full width at half maximum. Unless stated otherwise, all operations were performed using SPM12 default settings. Volume-to-volume rigid motion parameters (3 rotation parameters, 3 translation parameters) were estimated during the realignment step of the preprocessing and added as regressors to the individual first level models, as stated in the methods section on “First Level Modeling”.

#### First level modeling

First-level models contained regressors modeling the following conditions, as shown in Fig. 1: (1) The baseline script phase, (2) the inter-phase interval following the baseline script phase (“baseline thinking phase”), (3) the set-up phase, (4) the moral dilemma phase threatening protected values (“protected value script phase”) and (5) non-protected values (“non-protected value script phase”), (6) the question “How would you decide?”, (7) the inter-phase interval following this question, when a protected value was threatened (“protected decision-making phase”) and (8) when a non-protected value was threatened (“non-protected decision-making phase”), (9) the decision and rating recording phase. Six additional regressors modelled direct influences of subject motion, by including the three translation and three rotation parameters estimated during the realignment step of the preprocessing, without convolution by the hemodynamic response function. On the subject level, the following contrasts were calculated: protected value script phase vs. baseline script phase, non-protected value script phase vs. baseline script phase, protected decision-making phase vs. baseline thinking phase, and non-protected decision-making phase vs. baseline thinking phase.

#### Second level analysis

Individual contrasts between the protected and non-protected value script phases versus the baseline phase, and between the protected and non-protected decision-making phases versus the baseline thinking phase were entered into a full factorial model with within subject factors value (protected vs. non-protected) and phase (script vs. thinking), and between subject factor group (high-scorer versus low-scorer). The model included age as control-covariate of no interest. We calculated the following contrasts: protected > non-protected, non-protected > protected, high-scorers > low-scorers, low-scorers > high-scorers, and the corresponding interaction contrasts separately for both, the value script phase and the decision-making phase.

The resulting second-level T-images were analyzed by applying a single-voxel threshold of *p*_single_ < 0.001, and selecting only clusters with a family-wise-error (fwe) corrected cluster-level *p* value of *p*_fwe_ < 0.05, calculated using random-field theory methods implemented in SPM12 [25]. Activations were analyzed (and corrected for multiple comparisons) on the whole brain level.

## Results

### Behavioral data

**Fig. 2:**
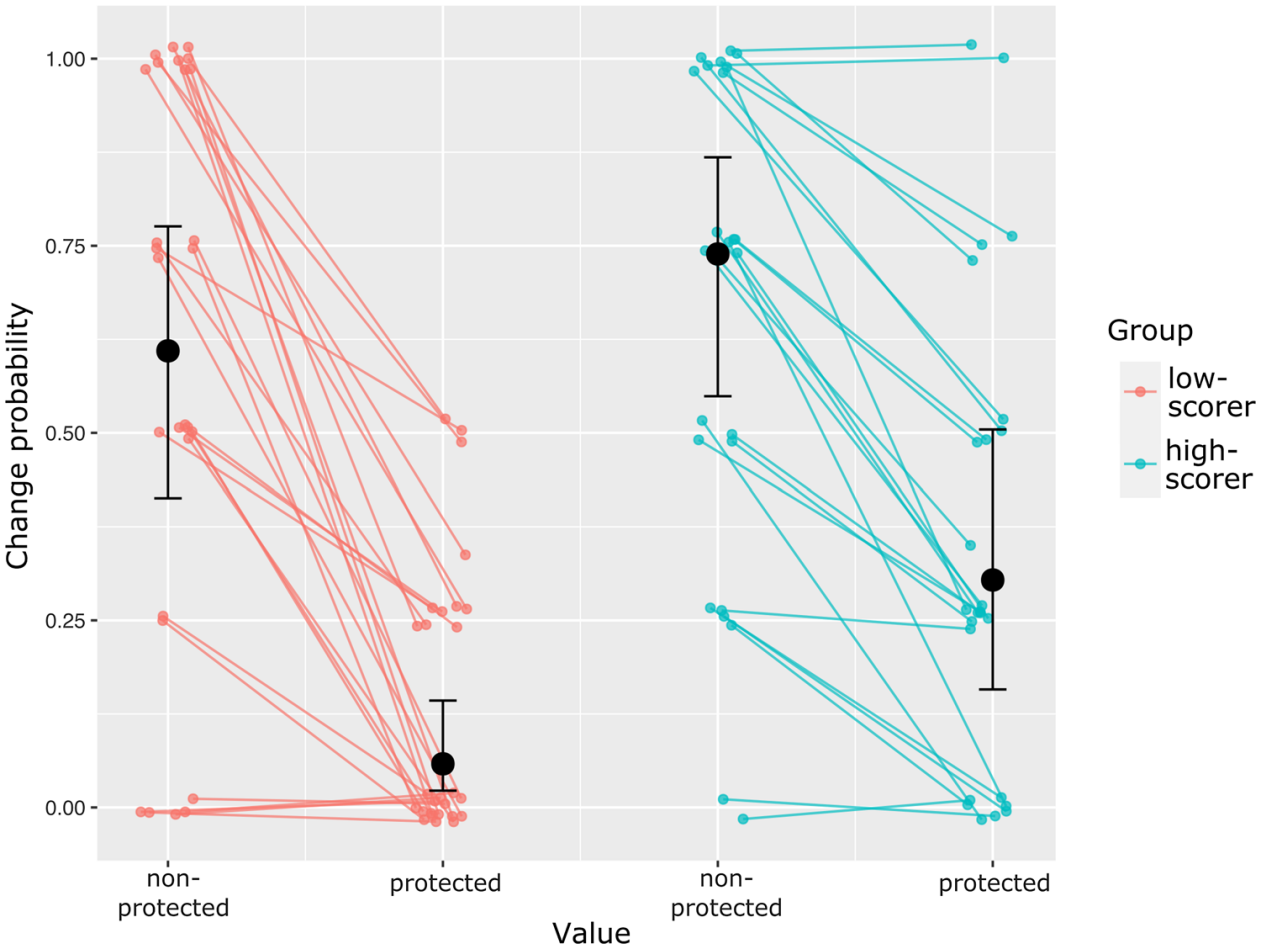
Marginal means for the probability of answering “yes” to the question “Would you change your decision for any amount of money?”, plotted for individual subjects (red: low-scorers, blue: high-scorers. Individual points were randomly jittered for better identifiability of individual subjects). Estimated marginal means and standard deviations from multi-level linear logistic regression model shown in black

Behavioral results are shown in Table 2. When presented with a protected value, participants were more likely to help the second person (*p* < 0.001), less likely to change their answer for any amount of money (*p* < 0.001), showed higher confidence in their decision (*p* < 0.001) and higher emotional involvement (*p* < 0.001), as compared to a non-protected value. High-scorers were less likely to help the second person (*p* = 0.023), and reported lower emotional involvement (*p* = 0.014). Older subjects reported lower emotional involvement (*p* = 0.020). There was a significant interaction between the type of moral value and the group, when subjects were asked if they would change their decision for any amount of money (*p* = 0.006): The difference between protected and non-protected moral values was significantly smaller within high-scorers, compared to low-scorers. Marginal means for this interaction are shown in Fig. 2.

**Table 2:** Multi-level linear regression models of behavioral data

	Decision^a^			Change^a^		
	Beta	95% CI^b^	*p*^c^	Beta	95% CI^b^	*p*^c^
Value^d^	3.31	(2.65; 20.70)	< 0.001*	− 3.23	(− 6.03, − 2.56)	0.001*
Group^e^	− 0.77	(− 2.10; 0.07)	0.023*	0.60	(− 0.68, 2.29)	0.206
Value × group	− 0.40	(− 16.93; 1.73)	0.371	1.36	(0.00; 3.83)	0.006*
Age	− 0.13	(− 0.80; 0.36)	0.296	0.32	(− 0.30, 1.08)	0.127

	Confidence^f^			Emotional involvement^f^		
	Beta	95% CI^b^	*p*^c^	Beta	95% CI^b^	*p*^c^
Value^d^	18.56	(12.29; 24.83)	< 0.001*	23.81	(15.74; 32.02)	< 0.001*
Group^e^	2.24	(− 6.97; 11.51)	0.557	− 10.67	(− 19.39; − 2.08)	0.014*
Value × group	− 8.11	(− 19.80; 3.37)	0.067	2.27	(− 8.59; 13.10)	0.664
Age	0.78	(− 2.72; 3.73)	0.620	− 4.12	(− 8.27; − 0.32)	0.020*

^a^Bivariate variables describing if subjects decided to help/if subjects would change their decision for any amount of money (1 = yes, 0 = no)

^b^Confidence interval between 2.5 and 97.5 percentiles, as determined by 10.000 bootstrapping iterations

^c^Determined by 10.000 random permutations of group and value labels

^d^Bivariate variable (1 = protected, 0 = nonprotected)

^e^Bivariate variable (1 = high-scorer, 0 = low-scorer)

^f^Ratings on a visual analog scale (0–100)

*Significant after correction for multiple comparisons using the Benjamini–Hochberg procedure with a false-discovery rate of 0.05

### Functional MRI data

#### Brain activation during value script phase

**Fig. 3:**
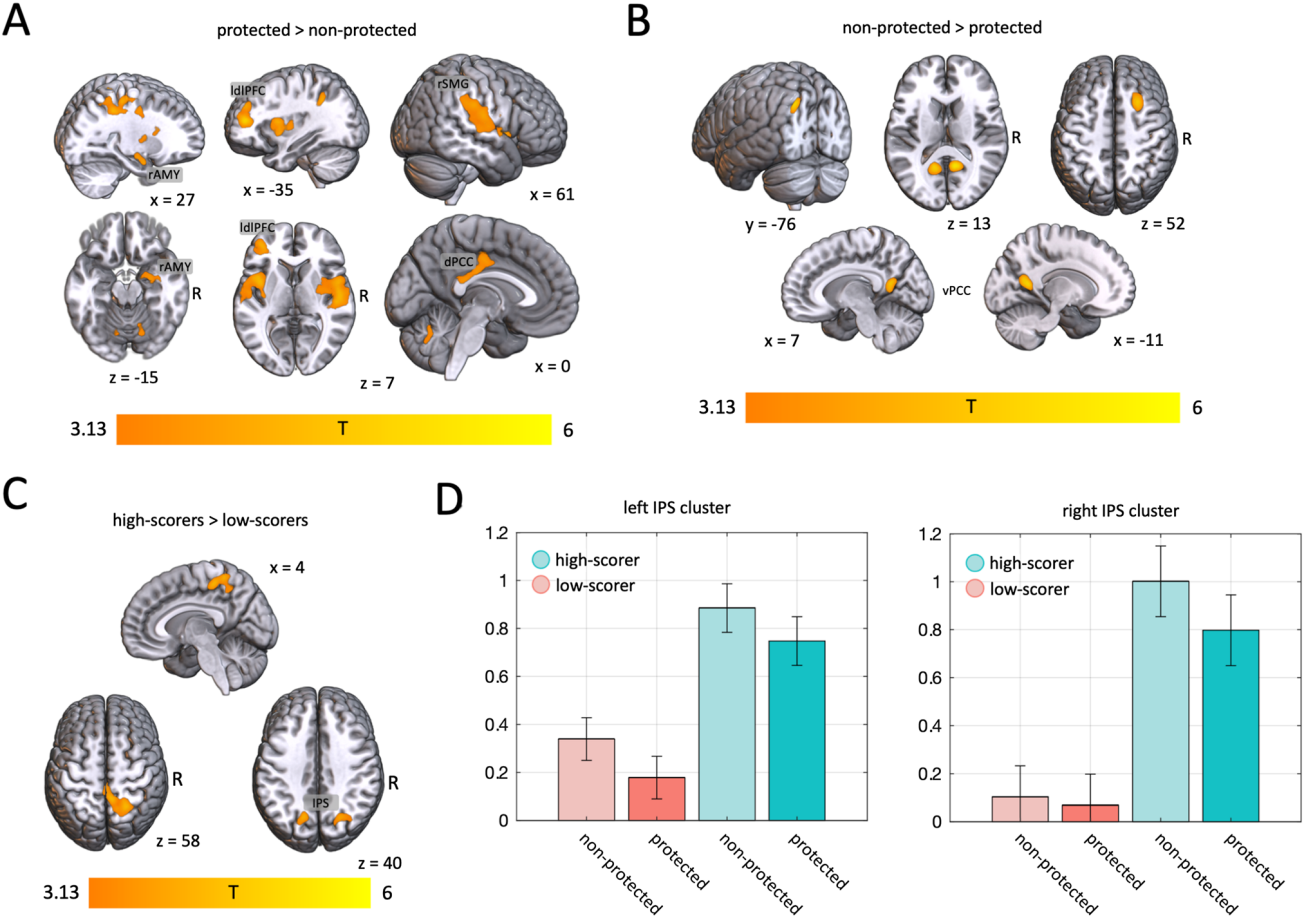
Functional imaging results. Statistical contrast of the BOLD response during the value script phase in the total sample, when an appeal to **A** a protected versus a non-protected value is made, and **B** vice versa. **C** Statistical contrast of the BOLD response during the decision-making phase, in participants scoring high versus low on the short Dark Triad scale. **D** Beta values extracted from the peaks of the bilateral IPS clusters shown in **C**. *rAMY* right amygdala, *ldlPFC* left dorsolateral prefrontal cortex, *rSMG* right supramarginal gyrus, *dPCC* dorsal posterior cingulate cortex, *vPCC* ventral posterior cingulate cortex, *IPS* intraparietal sulcus. The statistical maps resulting from the 2nd level analyses were visualized using MRIcroGL (https://github.com/rordenlab/MRIcroGL12; (Rorden & Brett, 2000)), using the spm152 anatomical template

Appeals for help in the context of protected values, compared to non-protected values, elicited significantly stronger blood-oxygenation level dependent (BOLD) responses in the total group in the following clusters: Left insula, secondary somatosensory, and auditory cortices (MNI_peak_ = (− 38, 4, − 4), *k* = 1488, *T*_peak_ = 4.73, *p*_fwe_ < 0.001). Right insula, secondary somatosensory cortex, auditory cortex, supramarginal gyrus, nucleus caudatus, dorsal posterior cingulate cortex (MNI_peak_ = (50, − 18, 4), *k* = 3858, *T*_peak_ = 4.87, *p*_fwe_ < 0.001). Right parahippocampal gyrus and amygdala (MNI_peak_ = (30, − 12, − 10), *k* = 233, *T*_*p*eak_ = 4.15, *p*_fwe_ = 0.005). Left dorsolateral prefrontal cortex and Broca’s area (MNI_peak_ = (− 38, 46, 12), *k* = 728, *T*_peak_ = 5.34, *p*_fwe_ < 0.001), as well as further somatosensory and motor regions. The resulting clusters are shown in Fig. 3A, and a cluster table is given as supplementary material S1.

Increases of the average BOLD response during appeals for help in the context of non-protected values, compared to protected values, were found in the following clusters: Left occipito-temporal junction (MNI_peak_ = (− 36, − 80, 38), *k* = 323, *T*_peak_ = 5.45, *p*_fwe_ = 0.012), right superior frontal sulcus (MNI_peak_ = (26, 20, 48), *k* = 500, *T*_peak_ = 5.36, *p*_fwe_ = 0.001), left ventral (retrosplenial) posterior cingulate cortex (MNI_peak_ = (− 12, − 54, 12), *k* = 344, *T*_peak_ = 5.20, *p*_fwe_ = 0.013), right ventral (retrosplenial) posterior cingulate cortex (MNI_peak_ = (12, − 54, 18), *k* = 344, *T*_peak_ = 5.90, *p*_fwe_ = 0.009). The resulting clusters are shown in Fig. 3B, and a cluster table is given as supplementary material S2.

No statistically significant effects of group or group x value interaction on BOLD responses during the value script phase were found.

#### Brain activation during decision-making phase

During the decision-making phase, participants scoring high on the Dark Triad showed an increased activation in the following clusters, compared to low-scoring participants: right superior parietal lobule and precuneus (MNI_peak_ = (22, − 54, 56), *k* = 962, *T*_peak_ = 5.14, *p*_fwe_ < 0.001), right intraparietal sulcus (IPS; MNI_peak_ = (24, − 70, 44), *k* = 390, *T*_peak_ = 4.45, *p*_fwe_ = 0.005), left IPS (MNI_peak_ = (− 16, − 66, 36), *k* = 242, *T*_peak_ = 4.44, *p*_fwe_ = 0.038). The resulting clusters are shown in Fig. 3C, box-plots of extracted beta values at the peaks of the bilateral IPS clusters are shown in Fig. 3D , a cluster table is given as supplementary material S3.

No statistically significant effects of value or group x value interaction on BOLD responses during the decision-making phase were found.

## Discussion

In this pilot study, we for the first time present data on the neural correlates of moral processing and moral decision-making following (imagined) ecologically valid requests for altruistic help, which directly contrast appeals to protected and non-protected values in subjects with high and low scores on Dark Triad traits.

The successful manipulation of the perceived type of moral value by different versions of the value script phase is corroborated by the significantly lower willingness of participants to change their decision for any amount of money. Our behavioral data show that charitable behavior was increased by appealing to protected versus non-protected values, as expected, and that protected values prompted increased emotional involvement, as well as increased confidence in the decisions taken.

In line with our hypotheses, the large and distributed network of regions, showing an increased activation by appeals to protected versus non-protected values, hints at multiple mechanisms involved: The increased activations in the bilateral insula and right amygdala are consistent with an increased emotional saliency of the situations appealing to protected values and stronger moral emotions [10, 26]. Activation of the right dorsolateral prefrontal cortex is consistent with the absolute, rule-based nature implied by protected values [13]. Finally, the increased activation within the right supramarginal gyrus is consistent with increased efforts to overcome emotional egocentricity, prompted by appeals to protected values [27].

In the opposite contrast (non-protected vs. protected values), regions in the bilateral ventral posterior cingulate cortex, as well as in the left occipito-temporal junction showed increased activations. These regions are associated to memory retrieval, self-referential and autobiographic processing [28], thus supporting a view in which the non-protected scenarios—which supposedly are encountered more often in daily life—activate related memory representations in the participants. The activation of a region in the right superior frontal sulcus, which is associated with the inhibition of automatic reaction tendencies and impulse control [29], is consistent with an increased effort to suppress an initial tendency to ignore the appeal to the non-protected value, which might not be required when protected values are appealed to, as these immediately prompt a rule-based decision.

In line with our hypotheses, participants with high scores on the short Dark Triad reported significantly lower emotional involvement and decided significantly less frequently to help the second person. Neuronally, they displayed increased activation during the decision-making phase. A large cluster including the right precuneus is consistent with an increased role of self-referential processes during decision-making and fits activations reported in a meta-analysis of functional correlates of psychopathy [30], one of the Dark Triad traits. Bilateral clusters in the intraparietal sulcus correspond to clusters found in a localizer task for utilitarian processing [13]. These regions were characterized in a computational imaging study as “accumulator regions”, which integrate cost and benefit signals up to a decision threshold, to form a behavioral decision in an economic decision-making paradigm [31]. Thus, these activations are consistent with an increased reliance on utilitarian cost–benefit calculations.

Older participants displayed reduced emotional involvement, which might be due to an increased serenity acquired during early adulthood, considering the group means of 26.7 years (low-scorers) and 22.9 years (high-scorers).

Crucially, a significant group × value interaction was found in the willingness of participants to change their decision for any amount of money: While protected values strongly suppress the willingness to change a decision for any amount of money in low-scorers, a substantial number of high-scorers maintains a high likelihood to change their decision for some amount of money, even when protected values are threatened. This directly demonstrates, that protected values do not supervene cost–benefit calculations to the same extent in high-scoring participants, as they do in low-scoring participants.

### Limitations

The following limitations must be considered: (1) Due to the relatively small sample size and the complexity of the task further studies are needed before firm conclusions can be drawn. (2) We restricted our analyses on the relationship between brain activations and the Dark Triad sum score which aggregates scores of three aspects: Machiavellianism, narcissism and psychopathy. Although in our study all three aspects highly correlated with the SD-3 sum score (*p* < 0.0001) the subjects receiving the exact same sum score may have actually differed in the intensity of the three aspects. Thus, in the supplement we present two tables, one presenting the results of the correlation analyses in the total sample and one showing the results of individual measurements on the three scales from all subjects (see supplementary material S4). (3) Our data is based on the imagination of complex situations involving moral decisions, as compared to experiencing such situations in real life. However, there is substantial evidence for the involvement of similar neural systems in imagined and actual movements [32, 33], inhibitory acts [34], and threatening scenarios [35]. Furthermore, narratives play a crucial role in the formation and sharing of moral values, both on a personal and cultural level [8], and script-driven imagery was successfully applied to study emotional processing in an ecologically valid fashion [23, 36]. (4) The high-scorers, defined by the highest quartile in this sample of students, extended the typical average scores reported from employers [37], graduates [38, 39] or competitive athletes [40]; however, to qualify for this group it was enough to collect 62.2% of the possible points in the scale suggesting that higher scores may exist in critical samples. As we considered only a male sample, we cannot draw conclusions on sex-specific differences in the processing of moral values. (5) The absence of interaction effects in BOLD responses might be due to saturation (floor and ceiling effects), resulting from large activation differences between high-scorers and low-scorers, as shown in Fig. 3D. These might be caused by our strategy of selecting only individuals with extreme SD3 scores. Future research including a representative population sample might increase the dynamic range of SD3 scores, and thereby enable analyses, which are more susceptible to interaction effects on a neuronal level. (6) This work built on the rich philosophical literature on the differences between protected and non-protected values. From a mere physiological perspective these scenarios may also differ in saliency consistent with differences in anterior insular and amygdalar activity, as these brain regions are implicated in the processing of salient stimuli.

## Conclusions

Data on the behavioral and neural level are consistent with the crucial role, which deeply culturally embedded moral values play in initiating altruistic behavior towards (vulnerable) others: They strongly activate brain circuits involved in moral emotions, social cognition and rule-based behavior compared to non-protected moral values. However, data further suggest that individuals scoring high on the Dark Triad are prone to keeping to utilitarian cost–benefit considerations also in situations signaling existential threat to others. Interindividual differences in the Dark Triad may impact individual decision making in current societal crises that touch protected moral values.

## Supplementary Information

Below is the link to the electronic supplementary material.

Supplementary file1 (PDF 187 KB)
