# Supplementary material for: Do I care for you or for me? Processing of protected and non-protected moral values in subjects with extreme scores on the Dark Triad
Source: Eur Arch Psychiatry Clin Neurosci. 2022 Oct 8;273(2):367–77. doi: 10.1007/s00406-022-01489-3 (PMC9547089; doi:10.1007/s00406-022-01489-3)
Supplement: Supplementary file 1 — Supplementary file1 (PDF 187 KB) [file 406_2022_1489_MOESM1_ESM.pdf]

Supplementary Table S1 Full-Factorial Analysis, whole brain results during the value script phase for the contrast protected > non-protected.

	k	***p***	***T***	***Z***	**MNI (peak voxell)**		
					x	***y***	***z***
Left Dorsolateral prefrontal cortex	728	<0.001	5.34	5.16	-38	46	12
			4.23	4.13	-32	42	28
			3.72	3.65	-24	26	16
Right precentral gyrus, postcentral gyrus	1102	<0.001	5.27	5.09	18	-32	58
			4.50	4.39	26	-42	48
			4.34	4.24	22	-46	74
Left postcentral gyrus	375	0.006	5.12	4.95	-20	-32	56
			3.78	3.71	-14	-48	76
			3.55	3.49	-22	-24	54
Right insula, auditory cortex, secondary somatosensory cortex, supramarginal gyrus, posterior cingulate cortex to nucleus caudatus, supplementary motor area	3858	<0.001	5.03	4.87	50	-18	4
			4.92	4.77	52	6	2
			4.83	4.69	-4	-14	38
Bilateral cerebellum	574	0.001	4.80	4.66	-22	-60	-42
			4.55	4.43	-14	-66	-42
			4.12	4.03	-28	-54	-44
Left insula, auditory cortex, secondary somatosensory cortex	1488	<0.001	4.73	4.60	-38	4	-4
			4.58	4.46	-38	4	12
			4.16	4.06	-50	4	2
Left sulcus postcentralis	341	0.001	4.57	4.45	-30	-46	22
			4.43	4.32	-30	-44	30
			4.33	4.23	-34	-50	36
Right gyrus parahippocampalis, amygdala	233	0.005	4.15	4.06	30	-12	-10
			4.14	4.05	26	-4	-20
			3.71	3.64	16	-8	-12

Supplementary Table S2 Full-Factorial Analysis, whole brain results during the value script phase for the contrast non-protected > protected

	k	***p***	***T***	***Z***	**MNI (peak voxell)**		
					x	***y***	***z***
Right retrosplenial cortex	344	0.009	5.90	5.66	12	-54	18
Left occipito-temporal junction	323	0.012	5.45	5.25	-36	-80	38
Right dorsolateral prefrontal cortex (BA9), superior frontal sulcus	500	0.001	5.36	5.17	26	20	48
Left retrosplenial cortex	320	0.013	5.20	5.03	-12	-54	12

Supplementary Table S3 Full-Factorial Analysis, whole brain results during the decision-making phase for the contrast high-scorers > low-scorers.

	k	***p***	***T***	***Z***	**MNI (peak voxell)**		
					x	***y***	***z***
Right superior parietal lobule, precuneus	962	<0.001	5.14	4.97	22	-54	56
			4.53	4.41	8	-46	60
			4.53	4.41	8	-30	52
Right intraparietal sulcus	390	0.005	4.45	4.34	24	-70	44
			3.96	3.88	16	-76	48
			3.37	3.32	36	-70	36
Left intraparietal sulcus	242	0.038	4.44	4.33	-16	-66	36

Supplementary Table 4a: Correlations between the SD3 sum score and the three aspects of the SD3 as well as among the three scales

		SD3_SUM	SD3_MA	SD3_NA	SD3_PP
D3_SUM	Pearson Correlation	1	.909**	.854**	.934**
	significance (2-sided)		0.000	0.000	0.000
	N	52	52	52	52
SD3_MA	Pearson Correlation	.909**	1	.620**	.805**
	significance (2-sided)	0.000		0.000	0.000
	N	52	52	52	52
SD3_NA	Pearson Correlation	.854**	.620**	1	.713**
	significance (2-sided)	0.000	0.000		0.000
	N	52	52	52	52
SD3_PP	Pearson Correlation	.934**	.805**	.713**	1
	significance (2-sided)	0.000	0.000	0.000	
		52	52	52	52

SD3: Short Dark Triad; D3_SUM: Total score; SD3_MA: Machiavellianism; SD3_NA: Narcissism Scale; SD3_PP: Psychopathy Scale

Supplementary Table 4b: SD3 individual scores

Participant	SD3 sum	SD3 psychopathy	SD3 narcissism	SD3 machiavellianism
1	50	13	21	16
2	89	28	25	36
3	54	9	21	24
4	87	24	28	35
5	51	12	15	24
6	102	27	32	43
7	51	10	20	21
8	92	31	31	30
9	44	12	12	20
10	93	25	35	33
11	51	12	27	12
12	90	23	27	40
13	51	11	24	16
14	97	30	38	29
15	50	11	20	19
16	92	25	33	34
17	48	10	21	17
18	85	19	39	27
19	46	11	25	10
20	89	20	42	27
21	49	12	22	15
22	86	23	27	36
23	55	11	22	22
24	53	13	24	16
25	88	21	35	32
26	54	12	23	19
27	90	23	36	31
28	46	13	19	14
29	85	25	32	28
30	54	13	21	20
31	84	20	31	33
32	51	11	24	16
33	84	23	31	30
34	55	12	23	20
35	86	14	38	34
36	114	35	43	36
37	55	13	24	18

38	85	27	29	29
39	53	12	21	20
40	88	25	29	34
41	55	13	23	19
42	91	28	32	31
43	55	10	26	19
44	93	30	33	30
45	56	9	28	19
46	87	22	35	30
47	53	10	24	19
48	93	22	33	38
49	55	13	23	19
50	87	22	32	33
51	48	10	18	20
52	56	13	18	25
